# Efficacy of intralesional injections of platelet‐rich plasma in patients with oral lichen planus: A pilot randomized clinical trial

**DOI:** 10.1002/cre2.550

**Published:** 2022-02-26

**Authors:** AbdelHameed Hijazi, Wesam Ahmed, Soheir Gaafar

**Affiliations:** ^1^ Department of Oral Medicine, Faculty of Dentistry Cairo University Cairo Egypt; ^2^ Department of Oral Medicine and Periodontology, Faculty of Dentistry Cairo University Cairo Egypt

**Keywords:** intralesional injections, oral lichen planus, platelet rich plasma

## Abstract

**Objective:**

To evaluate the clinical efficacy of intralesional platelet‐rich plasma (PRP) injections compared to intralesional triamcinolone acetonide (TA) injections in the treatment of erosive oral lichen planus (EOLP).

**Material and Methods:**

Twenty patients with EOLP were assigned randomly to either PRP or TA group. Patients received weekly intralesional injections for 4 weeks, and then followed up for 3 months on regular visits every 2 weeks. Pain scores using numerical pain score and clinical score were recorded by a blinded assessor each visit for all patients and remission score at the end of the trial was recorded.

**Results:**

Both groups showed significant improvement in the clinical parameters (pain and clinical score) “*p* = .001.” Regarding remission of the lesions, 80% of patients in the PRP group showed complete remission compared to 70% in the TA group. However, there is no statistical significance when comparing the two groups in pain score, clinical score, or remission.

**Conclusions:**

PRP injections could be considered as an effective alternative single treatment modality for EOLP. The protocol for this study registered in Clinicaltrials.gov registry under the identifier number: NCT03293368.

## 1 INTRODUCTION

Lichen planus is a chronic inflammatory mucocutaneous disease affecting adult patients, its prevalence between 0.22% and 5% of the worldwide population. Oral lichen planus (OLP) has multifactorial pathogenesis, including the involvement of both antigen‐specific (keratinocyte killing by CD8 T cytotoxic lymphocytes) and nonspecific mechanisms, as well as other mechanisms, such as mast cell degranulation and activation of matrix metalloproteinase. The chronic course of this potentially malignant disease could be explained by deficient antigen‐specific transforming growth factor‐beta 1 (TGF‐β1)‐mediated immunosuppression (Cheng et al., 2016; Sugerman et al., 2002).

The oral lesions of OLP are usually present in asymptomatic forms, but the atrophic‐erosive forms of OLP can cause a wide range of symptoms from mild discomfort to burning sensation up to severe pain, resulting in difficulty in speaking, eating, and swallowing. Patients with symptomatic OLP often require treatment to reduce signs and symptoms of the lesions (Thongprasom & Dhanuthai, 2008).

Different treatment modalities for OLP have been documented, such as corticosteroids, laser, antioxidants, topical anesthetic agents, and many more, but still, no treatment modality has been proven yet to be the single effective measure for controlling or curing the disease (Lodi et al., 2005).

Corticosteroids are considered the first‐choice pharmacological remedy for managing OLP lesions because of their anti‐inflammatory and immunosuppressive properties. Intralesional injections of corticosteroids were also used in managing OLP and proved effective. Although intralesional corticosteroids maintain a high concentration of the drug at the injected site, prolonged use is associated with many systemic adverse effects, such as bad taste, dryness of the mouth, candidal infection, mucosal atrophy, delayed wound healing, and in later stages, suppression of hypothalamus pituitary adrenal axis (Lee et al., 2013).

Due to the several side effects of the current treatments, there is a need to establish an effective and efficient treatment modality for erosive OLP with lesser or no adverse effects. Platelet‐rich plasma (PRP) is concentrated plasma of the patient's blood that contains a high concentration of platelets along with increased growth factors (GFs). PRP is emerging as an increasingly demanding clinical application as an alternative source of GFs for several dental procedures and oral mucosal lesions (Dhillon et al., 2012).

Activated platelets release various GFs, such as platelet‐derived GFs, transforming GF, fibroblast GF, and vascular endothelial GFs; these are the main contributing and leading factors for cell proliferation, differentiation, neoangiogenesis, toxins withdrawal, and cellular regeneration. PRP decreases the associated morbidity and promotes wound healing with anti‐inflammatory action. On the other hand, no adverse effects had been linked to PRP application and because it is usually prepared using the patient's own blood it does not elicit any immunological or allergic reactions (Pietrzak & Eppley, 2005).

Intraoral applications of PRP and PRF have been investigated in many fields, such as sinus elevation and ridge preservation where healing time, postoperative complications, bone quality, and volume were tested. But the results were inconsistent and the cumulative evidence shows insignificant differences between PCs and other comparable interventions (Bae et al., 2011; Del Fabbro et al., 2014).

The rationale for conducting this study was to provide a safe alternative treatment for patients suffering from resistant erosive OLP, which had proved a promising regenerative potential in the management of several refractory skin and mucosal lesions.

The aim of the present study was to evaluate the clinical efficacy regarding pain score and ulcer size of intralesional injections of PRP versus intralesional triamcinolone acetonide (TA) in the management of patients with erosive oral lichen planus (EOLP).

## 2 SUBJECTS AND METHODS

### 2.1 Study design and sitting

This pilot randomized controlled clinical trial was conducted at the faculty of dentistry, Cairo University, from September 2017 to December 2020.

### 2.2 Ethical approval

The study protocol has been approved (n. 17‐10‐7) by the research ethics committee (reference for Faculty of Dentistry, Cairo University).

### 2.3 Participants

#### 2.3.1 Eligibility criteria

##### Inclusion criteria

Patients with an age range from 18 to 70, presenting with a clinical picture that suggests the diagnosis of erosive OLP; bilateral, more or less symmetrical erosive lesions with a lacelike network of slightly raised gray‐white lesions (reticular pattern), and histopathological findings that confirms the diagnosis (liquefaction degeneration of the basal cell layer with irregular–saw teeth like rete pegs) are considered eligible for the study.

##### Exclusion criteria

Any patient suffering from systemic disorders, such as hematological diseases, severe cardiovascular diseases, or patients with platelet count <150,000/mm^3^; Hgb <11 g/dl, pregnant or active breastfeeding females, patients who had a lesion(s) with dysplasia, and patients who received treatment with any drugs that could cause oral lichenoid reactions, or receiving therapy with topical treatment for OLP in the last 2 weeks or systemic treatment for OLP in the past 3 months, anticoagulants or immunosuppression drugs are excluded.

### 2.4 Interventions

PRP was prepared at the oral medicine department clinic from autologous venous blood collected in the same visit according to Mostafa et al. (2013). In the control group, TA 40 mg/1 ml aqueous suspension (Synthecortin Ampoule; *Medical Union Pharmaceuticals MUP.; Egypt*) was used (Lee et al., 2013).

The patients in both groups had received intralesional injections once every week for 4 weeks. The injections in both groups were applied after a field block with Mepevicaine 3% *Alexandria Co.; Egypt* anesthetic without vasoconstrictor. An amount of 0.5 ml of each treatment was injected per 1 cm^2^ of ulcerated mucosa using a 25‐gauge needle.

After the fourth week of intralesional TA application in the control group, topical Miconazole oral gel was prescribed for 1 week three times per day.

### 2.5 Outcomes

#### 2.5.1 Primary outcome

The pain was self‐assessed by the patient using an 11‐point (0–10) numerical rating scale, in which (0 = no pain) and (10 = the worst possible pain) (Seymour, 1982).

#### 2.5.2 Secondary outcomes

1. Clinical picture assessed by Thongprasom sign scoring (Thongprasom et al., 1992), the measures were recorded using a periodontal probe measuring the highest length and width of the lesion giving an average area space as follows:
  Score 0 = no lesions, normal mucosa
  Score 1 = mild white striae, no erythematous area
  Score 2 = White striae with an atrophic area less than 1 cm^2^
  Score 3 = White striae with an atrophic area more than 1 cm^2^
  Score 4 = White striae with an erosive area less than 1 cm^2^
  Score 5 = White striae with an erosive area more than 1 cm^2^
2. Remission time according to Conrotto et al. (2006), the measures were recorded using a binary scale ([yes/stable or no/not stable]: yes indicates signs score more than 1; no indicates signs score equals 1 or less).

The main investigator H. A. and the assessor who was blinded recorded outcomes before the treatment as baseline data and at the beginning of each visit using the previous scale and scores.

### 2.6 Sample size

Based on a previous study by Xia et al. (2006), a total sample size of 8 (4 in each group) will have 90% power to detect a difference in the VAS means between the two groups using *t*‐test with a 0.05 two‐sided significance level. The number is increased to a total sample size of 10 to allow for the use of a nonparametric test. The sample was further increased to 20 (10 participants in each group) to allow dropout loss. Sample size estimation was performed by nQuery statistical package.

### 2.7 Sequence generation

Simple randomization using computer‐based sequence generation software was used after patients' consent of enrollment.

### 2.8 Allocation concealment mechanism

This trial used no concealment, as the interventions cannot be blinded from the patients or the investigator (too obvious to know).

### 2.9 Implementation

The main investigator H. A. was responsible for the sequence generation, allocation, and enrollment of the participants.

### 2.10 Blinding

The assessor of outcomes was blinded; she was NOT involved in any step during patients' allocation or during treatment delivery.

### 2.11 Statistical methods

The mean and standard deviation values were calculated for each group in each test. Data were explored for normality using Kolmogorov–Smirnov and Shapiro–Wilk tests, and showed nonparametric distribution. Mann–Whitney test was used to compare between two groups in nonrelated samples. Wilcoxon test was used to compare between two groups in related samples. The significance level was set at *p* ≤ .05. Statistical analysis was performed with IBM® SPSS® Statistics Version 20 for Windows.

## 3 RESULTS

### 3.1 Participant timeline

A biopsy of the lesion was performed for histopathologic examination for the confirmation of the diagnosis of new undiagnosed patients. Then, recruited patients were assigned to one of the groups (intervention or control), after that, all participants in the intervention group had provided a recent complete blood picture to confirm that their platelets count is over 150,000, and pretreatment records were obtained for the lesions. Then, each week the same treatment protocol for each group was followed for consecutive 4 weeks; in total four injections were injected for each participant. After the last injection, the patients were followed up for 1 week to obtain the endpoint measures. The main investigator H. A. and the assessor followed up with all participants every 2 weeks for 3 months from the last visit to report flare episodes of the disease.

### 3.2 Recruitment

The patient's database from the department of oral medicine and periodontology was filtered by their condition; eligible subjects were contacted to ask for a follow‐up visit. Those who agreed to be enrolled in the trial were asked to sign an informed consent, and then all participants were allocated randomly to either study group according to the computer‐generated sequence.

The present study included 20 patients divided into two groups (10 in each group) suffering from EOLP, with the age range 24–65 years. Group (A) received intralesional PRP injections, while Group (B) received intralesional TA injections. Both treatments were administered once a week for four consecutive weeks. Photographs for the oral lesions, numerical pain score, and measuring of ulcer size were registered at baseline and at every visit after. The patients were followed up after treatment every 2 weeks for 3 months. The remission of the clinical outcomes was recorded at the end of the follow‐up period.

**Figure 1:**
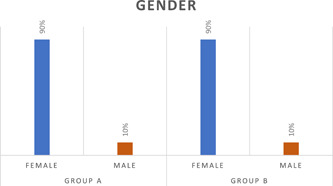
Bar chart representing gender distribution frequency for different groups

Regarding mean age and gender distribution, there was no statistically significant difference between Group (A) and Group (B) where (*p* = .098). Mean age distribution in groups is shown in Table 1. Figure 1 presents gender distribution frequency among study groups, where both groups included nine female patients and one male patient. Concerning pain scores the only significant statistical difference was noted at Week 4 (end of treatment) between both groups where (*p* = .010), also similar results were recorded in clinical scores where (*p* = .042). Where at Week 17 both groups showed significant reduction in the scores and there was no statistically significant difference when compared to each other.

**Table 1:** Age distribution among groups

Variables	Age			
	Mean	SD	Min	Max
Group A	42.60	11.46	24.00	59.00
Group B	50.30	7.94	40.00	65.00
*p*‐value*	.098 ns			

Abbreviation: ns, nonsignificant.

*Significant (*p* < .05).

**Figure 2:**
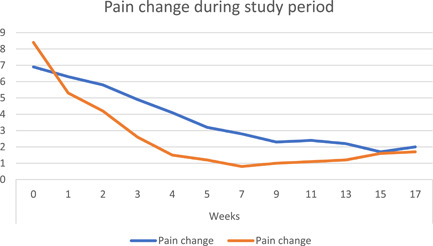
Line graph shows mean pain score change in both groups during the study period

**Figure 3:**
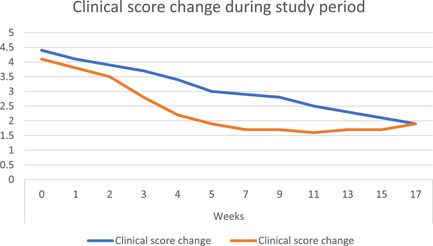
Line graph shows mean clinical score change in both groups during the study period

Figures 2 and 3 show a line graph of changes in pain and clinical scores through the trial period.

Remission score after 3 months of follow‐up showed no statistically significant difference between Group (A) and Group (B) where (*p* = .615).

**Figure 4:**
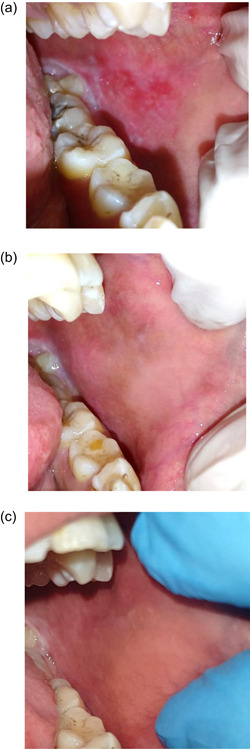
(a) A clinical photo showing irregular erosion affecting left buccal mucosa surrounded with white interlacing keratotic lesion resembling Wickham Striae. (b) After 3 weeks of treatment with intralesional platelet‐rich plasma (PRP) injections, the lesion shows complete remission with mild keratotic striae. (c) After 17 weeks of follow‐up, the lesion in remission

**Figure 5:**
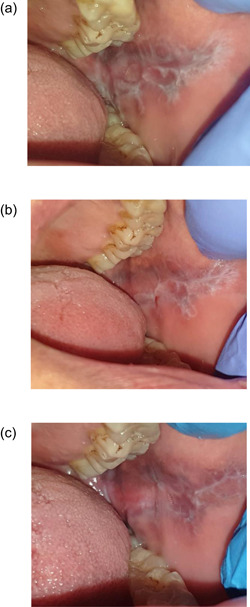
(a) A clinical photo showing irregular erosion affecting left buccal mucosa surrounded with white interlacing keratotic lesion resembling Wickham Striae and hyperpigmented mucosa. (b) After 3 weeks of treatment with intralesional triamcinolone acetonide (TA) injections, the lesion shows complete remission with keratotic striae. (c) After 17 weeks of follow‐up, the lesion still in remission

Figure 4a,b,c shows clinical photos of EOLP lesion treated with intralesional injections of PRP at baseline, 4 weeks, and 17 weeks interval, respectively. Figure 5a,b,c shows clinical photos of EOLP lesion treated with intralesional injections of TA at baseline, 4 weeks, and 17 weeks interval, respectively.

## 4 DISCUSSION

Since OLP is a chronic disease, a complete cure is very difficult to achieve. Topical steroids are the mainstay of palliative therapy of symptomatic OLP. Due to the lack of strong evidence to support the single usage of any treatment for symptomatic OLP lesions, alternative remedies are frequently considered. With inconsistent therapeutic efficacies, many drugs and interventions have been investigated in symptomatic OLP with an urge to get more tolerable and safe treatment (Thongprasom et al., 2013).

GFs including PDGF, TGF‐β, EGF, IGF, fibronectin, and VEGF play a pivotal role in all phases of healing of the tissue by recruiting mesenchymal cells and also during the synthesis of the extracellular matrix (Dionyssiou et al., 2013). Interestingly, TGF‐β (released from platelet alpha‐granules) is found to be an essential differentiation factor in the homeostasis as well as in the development of T regulatory (T regs), which further inhibit inflammation and autoimmunity by counteracting the effects of other T helper cells, also there was strong evidence of TGF‐β pathway blockage by increased expression of Smad‐7 in erythematous OLP (El‐Komy et al., 2015; Karatsaidis et al., 2003).

PRP has been a breakthrough since the early 80 s in the stimulation and acceleration of soft tissue healing. Several clinical studies had reported promising results using PRP as a treatment for several disorders in different routes. Platelets release a cocktail of GFs, which are believed to mimic the physiological healing process through nuclear factor‐kappa beta (NF‐κβ) suppression (Chakravdhanula et al., 2016).

All participants in the present study were free from any systemic disease that compromise the diagnosis of idiopathic OLP, nor taking drugs that affected platelet function. The patients had complete blood picture at baseline. Only patients with platelet counts ≥150,000/µl were included in the study. One of the main factors affecting platelets number in PRP is baseline platelets count in the whole blood (Andrade et al., 2008).

The response to PRP injections was variable among participants in the present study, which required the increase of treatment periods. It was noted that tongue lesions had a slower reduction in both pain and clinical scores within the first 4 weeks of treatment; fluctuations of the symptoms in a few cases in the PRP group were recorded so intralesional injections were continued every 2 weeks of follow‐up until 3 months in those cases. While other clinical trials reported different clinical responses, and they selected different treatment intervals for intralesional injections of PRP (Sethi Ahuja et al., 2020; Sobhy et al., 2020). Sobhy et al. (2020) applied intralesional PRP injections for five sessions with 2 weeks interval with no side effects or lesion recurrence; Sethi Ahuja et al. (2020) reported significant lesion size reduction on the eighth week of treatment with intralesional PRP injections when selecting a treatment plan of weekly injections for 8 weeks.

More recently, Bennardo et al. (2021) reported no statistically significant difference in pain scores after 8 weeks of intralesional injections of injectable platelet‐rich fibrin (i‐PRF) compared to intralesional injections of TA for treatment of symptomatic OLP.

Our results had shown a significant reduction of pain scores in both groups after 3 months of follow‐up after treatment. In addition, there was a statistical difference between Group (A) and Group (B) at 4 weeks period in terms of pain reduction with no statistically significant difference when comparing both groups at the end of the study. These findings are in accordance with the previous work of Sobhy et al. (2020) who used PRP intralesional injections for recalcitrant EOLP patients.

Pain reduction in EOLP patients after intralesional PRP or i‐PRF treatment was significant after 2 months (Bennardo et al., 2021; Sethi Ahuja et al., 2020) and 3 months of follow‐up (Sobhy et al., 2020). Also, these results were similar to Loré et al. (2016) and Merigo et al. (2018) where they had reported significant symptoms reduction after 8 weeks of topical PRP application in erosive OLP, despite different frequencies of application (weekly and daily application, respectively) they showed the efficacy of PRP treatment for erosive OLP.

Regarding the clinical scores in each group at the end of the trial, there was no statistical difference between the two groups. However, a significant statistical difference was observed in clinical score between Group (A) and Group (B) at Week 4, also when comparing clinical score in Group (A) between Week 4 and 17, but no statistical difference in Group (B) was noted. This implies a slower clinical response but a steady effect of PRP injections than TA injections. This goes in agreement with previously published literature about reduced skin and mucosal ulcers size in different diseases using intralesional PRP at different intervals and different end of study visits ranging from 8 weeks to 24 months (El‐Komy et al., 2015; Martínez‐Zapata et al., 2009, 2012).

Previous work done by Loré et al. (2016) on topical application of PRP for EOLP lesions in four patients showed complete response in 50% of the lesions after 8 weeks of weekly interval treatment. His work is not in accordance with the results of the present study due to the difference in the methodology “topical application” and duration of the study period. Interestingly, a case report for one patient with recalcitrant EOLP showed significant improvement of clinical response after 8 weeks of daily application of topical mouth rinse of PRP by Merigo et al. (2018). These results indicate a possible clinical effect of topical PRP application for EOLP but lack of standardized treatment protocol, which did not match the results of recent work of intralesional injections of PRP reported by Sethi Ahuja et al. (2020) and Sobhy et al. (2020).

Recently, significant clinical score improvement was reported after intralesional PRP application in EOLP, where Sethi Ahuja et al. (2020) have reported 91.66% erythema reduction of the lesions, while Sobhy et al. (2020) reported significant reticulation/keratosis, erythema, and ulceration (REU) score improvement with no side effects.

In the present study, no significant statistical results were recorded between groups regarding remission score after 3 months of treatment, while El‐Komy et al. (2015) reported longer remission when using intralesional PRP for treating oral erosions in PV patients. Orlandi et al. (2018) reported long‐term remission in a case report of ulcerative skin lichen planus treated with autologous skin graft with intralesional PRP injections followed up for 4 years. As well as, long‐term remission have been reported after 2, 3 months of follow‐up for patients with EOLP treated with intralesional injections of PRP (Sethi Ahuja et al., 2020; Sobhy et al., 2020) respectively.

### 4.1 Study limitations

The small sample size and relatively short follow‐up period are the main limitations of the current pilot study. Moreover, challenges in standardization of baseline measurements as lesion site, size, and pain score were also found during patients' recruitment. These limitations may have affected the overall estimation of the PRP injections healing effect.

## 5 CONCLUSIONS

Based on the findings of this study it can be concluded that intralesional PRP injections can reduce subjective pain and objective clinical scores when used as an alternative treatment modality for refractory lesions of EOLP. In addition, intralesional PRP injections are safer in the treatment of EOLP lesions for the patient to spare long‐term side effects of corticosteroids. Further randomized clinical trials with larger sample size and longer follow‐up period on the safety of PRP injections on EOLP (as a potentially malignant disease) are recommended.

## CONFLICT OF INTERESTS

The authors declare no conflict of interest.

## AUTHOR CONTRIBUTIONS

W. Ahmed and S. Gaafar designed and supervised the study. A. Hijazi conducted sample recruitment, treatment delivery, data collection, and results analysis. All authors read and approved the final manuscript.

## Data Availability

The data that support the findings of this study are available from the corresponding author upon reasonable request.
